# The Role of Ergothioneine in Red Blood Cell Biology: A Review and Perspective

**DOI:** 10.3390/antiox13060717

**Published:** 2024-06-13

**Authors:** Tiffany A. Thomas, Richard O. Francis, James C. Zimring, Joseph P. Kao, Travis Nemkov, Steven L. Spitalnik

**Affiliations:** 1 Laboratory of Transfusion Biology, Department of Pathology and Cell Biology, Columbia University Irving Medical Center, New York, NY 10032, USA; tt2254@cumc.columbia.edu (T.A.T.);; 2 Department of Pathology, University of Virginia School of Medicine, Charlottesville, VA 22903, USA; 3 Center for Biomedical Engineering, Department of Physiology, University of Maryland School of Medicine, Baltimore, MD 21201, USA; 4 Department of Biochemistry and Molecular Genetics, University of Colorado Anschutz Medical Campus, Denver, CO 80203, USA

**Keywords:** ergothioneine, antioxidant, RBC, erythrocyte, ROS, nutraceutical, supplement

## Abstract

Oxidative stress can damage tissues and cells, and their resilience or susceptibility depends on the robustness of their antioxidant mechanisms. The latter include small molecules, proteins, and enzymes, which are linked together in metabolic pathways. Red blood cells are particularly susceptible to oxidative stress due to their large number of hemoglobin molecules, which can undergo auto-oxidation. This yields reactive oxygen species that participate in Fenton chemistry, ultimately damaging their membranes and cytosolic constituents. Fortunately, red blood cells contain robust antioxidant systems to enable them to circulate and perform their physiological functions, particularly delivering oxygen and removing carbon dioxide. Nonetheless, if red blood cells have insufficient antioxidant reserves (e.g., due to genetics, diet, disease, or toxin exposure), this can induce hemolysis in vivo or enhance susceptibility to a “storage lesion” in vitro, when blood donations are refrigerator-stored for transfusion purposes. Ergothioneine, a small molecule not synthesized by mammals, is obtained only through the diet. It is absorbed from the gut and enters cells using a highly specific transporter (i.e., SLC22A4). Certain cells and tissues, particularly red blood cells, contain high ergothioneine levels. Although no deficiency-related disease has been identified, evidence suggests ergothioneine may be a beneficial “nutraceutical.” Given the requirements of red blood cells to resist oxidative stress and their high ergothioneine content, this review discusses ergothioneine’s potential importance in protecting these cells and identifies knowledge gaps regarding its relevance in enhancing red blood cell circulatory, storage, and transfusion quality.

## 1. Introduction

Red blood cells (RBCs) are critically important for organismal biology by, for example, delivering oxygen to multiple tissues and removing carbon dioxide [1]. To transport oxygen, every human RBC contains ~250 million hemoglobin molecules [2], each of which contains four heme groups, with each of those containing one atom of the ferrous form of iron caged within a porphyrin ring. Because oxygen binds directly to ferrous iron in hemoglobin, oxygen-rich hemoglobin is at risk of undergoing auto-oxidation, whereby oxygen extracts an extra electron, thus converting ferrous iron (i.e., Fe^+2^) to its ferric (i.e., Fe^+3^) state, which yields met-hemoglobin, while simultaneously producing superoxide. The latter sparks a series of reactions, exacerbated by the abundance of iron inside RBCs, to produce multiple reactive oxygen species (ROS) and other toxic metabolites, including the hydroxyl radical and hydrogen peroxide. Thus, during their circulatory lifespan (e.g., ~120 days in humans), RBCs are continuously exposed to significant oxidative stresses as a result of their normal biological function. Therefore, to protect their cytosolic components and plasma membrane integrity, and to reduce non-oxygen-transporting met-hemoglobin back to its functional form, evolution provided RBCs with multiple mechanisms to protect themselves from oxidative stress. These include small molecules (e.g., vitamin C, vitamin E, reduced glutathione (i.e., GSH)) and proteins (e.g., superoxide dismutase, glutathione peroxidase, catalase, peroxiredoxin 2), which can eliminate ROS by participating in specific metabolic pathways. In addition, multiple redundant pathways repair RBC lipids and proteins damaged by oxidant stress (for reviews, see [3,4,5]), and enzymes that maintain antioxidant capacity continually replenish these pathways (e.g., glutathione reductase). To this end, the relevant small molecules, proteins, enzymes, reactions, and metabolic pathways have been studied intensively for decades in an effort, not only to understand how RBCs experience and respond to oxidant stress in health and disease, but also to develop approaches to enable RBCs to avoid and resist oxidant stress and enhance their abilities to ameliorate it [6].

**Figure 1:**
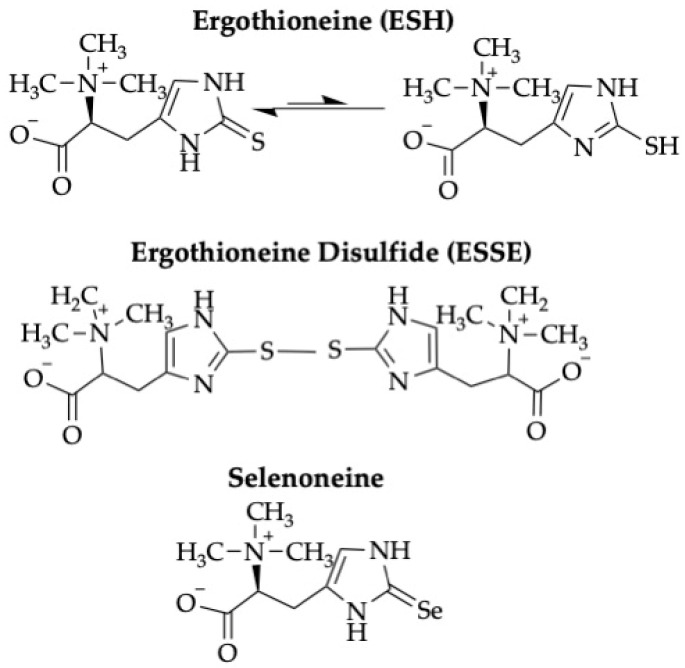
**Structure of ergothioneine and related compounds.** ESH has two tautomeric forms—thione (on the left) and thiol (on the right). Under physiological conditions, ESH primarily exists as its thione tautomer. The disulfide form, ESSE, is produced by the oxidation of ESH. Selenoneine, a selenium-containing analogue of ESH, is abundant in certain marine animals, is found in human RBCs, and may function as an antioxidant.

Among the myriad independent, overlapping, and redundant pathways that handle oxidant stress in RBCs, an interesting and unusual molecule was identified more than a century ago: ergothioneine (i.e., ESH; see Figure 1) [7]. Its primary function appears to be as an antioxidant, and it is found in multiple organisms, tissues, and cells. Yet, despite many studies of its structure, location, and function, multiple mysteries still remain. Although it is the subject of excellent general reviews [8,9,10], this current contribution focuses on its potential relevance and importance for mammalian, primarily human, RBC biology, for which, to the best of our knowledge, no specific and comprehensive review has yet been published.

ESH is not synthesized by humans or other mammals, but rather, is incorporated from multiple dietary sources, including mushrooms, which have particularly high amounts (for a general review, see [11]). Nonetheless, high levels of ESH are found in multiple human tissues, including mature RBCs; this finding, along with the existence of a transporter, SLC22A4, which transports ESH with high specificity (see below), suggests that ESH has significant biological importance. However, no known disease-inducing deficiency states have yet been identified in humans or in animal models, although, even when ESH levels are “undetectable” in various animal models (see below), it is not clear that the compound is completely absent from the relevant tissues. Nonetheless, because no such deficiency disease has yet been identified, ESH is not classified as a “vitamin”, although it may be characterized as a “nutraceutical” (see below). Indeed, given its apparent safety as a dietary supplement for humans [12,13], it has been proposed to have clinical utility in various settings [10], including aging [14,15], cognitive dysfunction [16], and infection [17], thereby suggesting its potential relevance as a nutraceutical. Its efficacy in such settings has been demonstrated in animal models, as expanded on below.

## 2. ESH in the Diet and Dietary Supplementation

ESH is not synthesized de novo by animals [18], but rather by fungi [19], principally certain species of mushrooms (e.g., king bolete and oyster mushrooms), although it can also be found in lower concentrations in multiple other foods including beans (e.g., black turtle beans, red kidney beans), grains, eggs, and meat [10,20]. In addition to ESH being obtained by dietary means, purified preparations of ESH are available that are suitable for human use [21].

Dietary supplementation with ESH has been studied in various animal models. For example, in mice, it increases levels in “whole blood” and other tissues [22]. In rats supplemented with tritium-labeled ESH, this compound becomes rapidly detectable in plasma and then undetectable; however, its levels are stable in “corpuscles”, presumably RBCs (see below) [23]. In addition, little, or no, tritiated ESH disappeared from the blood in vivo after 1 week of fasting [23]. Moreover, dietary ESH in rats protected their kidneys and liver against Fenton reaction-derived oxidative damage, particularly against lipid peroxidation [24].

The initial studies exploring ESH dietary supplementation in humans, particularly in the context of a nutraceutical, examined ESH amounts in various foods [11,25] and focused on healthy human volunteers. For example, one human clinical trial demonstrated the bioavailability of ESH from eating mushrooms [26]. Another showed the “slow” accumulation of pure ESH into “whole blood”, in contrast to plasma, consistent with the possible incorporation of ESH into RBCs during erythropoiesis [27]. To begin evaluating the potential therapeutic benefits of pure dietary ESH, another human clinical trial found that plasma levels increased, and then decreased rapidly, whereas RBC levels began increasing ~7 days after initiating ESH administration, again consistent with its incorporation during erythropoiesis [27]. This study also found decreasing trends in oxidative stress and inflammation, suggestive of a therapeutic effect [27]. More recently, a description of the design of the “first” randomized human clinical trial was published, which aims to study the therapeutic effects of ESH supplementation in patients with the metabolic syndrome [28]. The goal of this “pilot” trial is to understand the magnitude of any potential effects, thereby allowing for power calculations to be determined for a subsequent definitive trial [28]; however, this trial has not yet enrolled any patients [29].

Even though no therapeutic benefits have been proven to date in humans, dietary supplementation with ESH seems safe for human use. For example, regulatory agencies in Europe [12] and the United States [13] have deemed it safe as a dietary supplement or food additive, at least up to 30 mg/day for adults and 20 mg/day for children. Indeed, this even seems to hold for fetuses and breast-feeding neonates [30].

Nonetheless, some caution is required when developing inclusion and exclusion criteria during the design of clinical trials, because there is, at least, the suggestion that ESH may exacerbate cancer and certain infections (e.g., tuberculosis) by preventing the oxidative stress that may be protective in these pathogenic processes [8]. In addition, ESH supplementation may affect immune function via the potentiation of toll-like receptor (TLR) signaling or through other mechanisms [20,31]. Thus, potential adverse effects should be considered when designing clinical trials of dietary supplementation with ESH in specific clinical settings.

Finally, several recent studies examined selenoneine, a selenium-containing molecule that is structurally similar to ESH (Figure 1) and may have similar biological effects. Selenoneine is present in high amounts in the skin of beluga whales and in RBCs among the Inuit who consume this food [32]. By analogy with ESH, selenoneine may function as an antioxidant. Although it appears to be transported into human cells by SLC22A4, the ESH transporter (see below) [33], and may be important in protection against methylmercury toxicity, its biology is still not well understood.

## 3. ESH Mechanism(s) of Action: What Does It Do and How Does It Work?

### 3.1. ESH Detoxifies ROS and Binds Metal Cations to Protect Cells from Damage

At a molecular level, a major mechanism by which ESH exerts its antioxidant function(s) involves the direct inactivation of various ROS and free radicals [10], including the hydroxyl radical [34] and singlet oxygen [35], the latter of which can be produced by neutrophils during inflammatory processes [36]. In contrast, ESH does not seem to react directly with hydrogen peroxide or superoxide anion [37,38]. Moreover, although it may not directly detoxify hydrogen peroxide [37,38], ESH can protect cells from hydrogen peroxide-mediated damage [39]. In addition, ESH, which can alternate between its tautomeric forms (i.e., thiol or thione (Figure 1)), may primarily exist as a thione inside cells; as such, it is protected from auto-oxidation, making it more stable than GSH, which, as a thiol, is readily oxidized and, thereby, inactivated [39].

Interestingly, ESH can also directly interact with various metal cations, potentially including iron [34]. Thus, in a physiological way, ESH may enhance ferrochelatase-mediated iron incorporation into protoporphyrin IX to produce heme during normal hemoglobin synthesis, perhaps by helping maintain iron in its reduced, ferrous (Fe^+2^) state [40]. In addition, ESH may neutralize highly reactive ferryl (Fe^+4^) hemoglobin [38], thereby preventing met-hemoglobin production and free radical-catalyzed lipid peroxidation [41]. Given the close homology between myoglobin in muscle cells and hemoglobin in RBCs, and their corresponding roles in oxygen delivery, it is perhaps not surprising that ESH can also reduce the ferryl (Fe^+4^) form of myoglobin [37]. As one potential clinical application of ESH’s interactions with metal cations, it ameliorated iron-induced liver toxicity in rats, potentially due to its ability to scavenge ROS and potentially bind iron [42].

### 3.2. How Is ESH Regenerated after Its Oxidation to the ESSE Disulfide?

**Figure 2:**
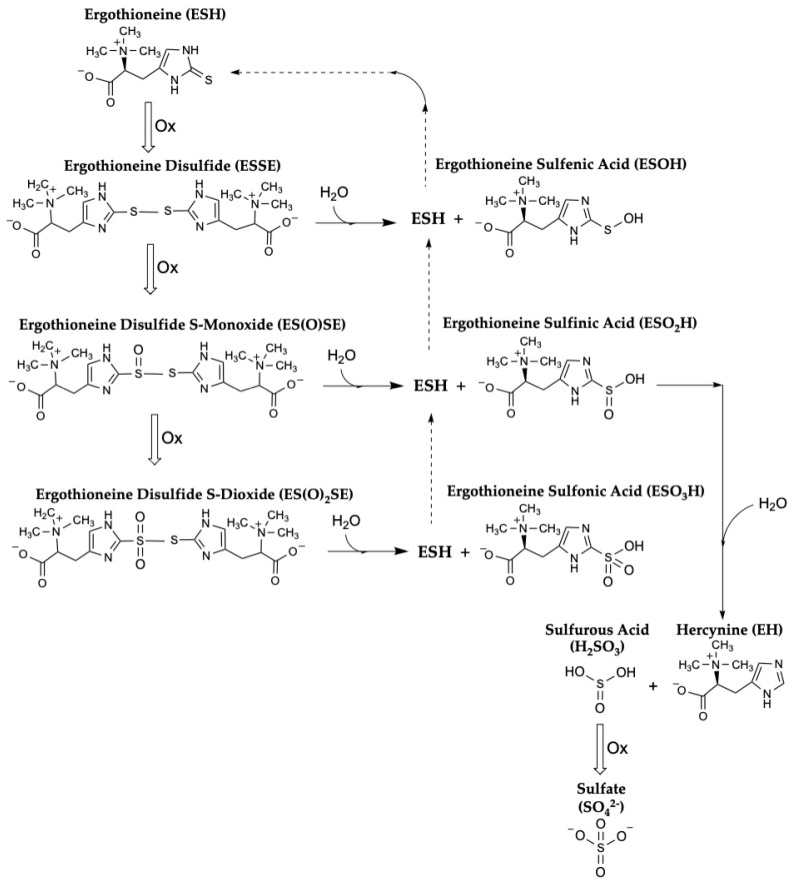
**One proposed pathway of ESH oxidation with subsequent regeneration of reduced ESH.** This pathway does not require reducing equivalents provided by NADPH (figure inspired by Ref. [44]). Nonetheless, other pathways have been proposed, including ones involving GSH to regenerate reduced ESH [46,47,49,51].

Some uncertainty remains regarding the exact metabolic pathways and reaction mechanisms by which ESH exerts its antioxidant effects and is then regenerated and/or catabolized [22,43]. For example, Servillo et al. state that “…in the presence of oxidants, ESH can form the disulfide according to the usual pattern, but this disulfide (ESSE) can behave very different from alkylthiol disulfides, in that, being unstable at physiological pH, it undergoes a progressive decomposition by disproportion. Our data show that, from 2 mol of ESSE, 3 mol of ESH and 1 mol of EH are formed. *Notably, the partial ESH regeneration from ESSE does not require reducing substances, the process being a disproportion*” (emphasis added; Figure 2) [44]. Therefore, these results suggest that providing reducing equivalents, such as NADPH derived from the pentose phosphate pathway, may not be required to maintain reasonable amounts of the reduced form of ESH in cells. Nonetheless, the slow kinetics of this disproportionation reaction raises questions about its biological relevance. In addition, these authors suggest that this process could occur inside cells, not just in vitro, as when exposing endothelial cells to oxidative stress caused by high glucose levels, paraquat, superoxide, or hydrogen peroxide [45], although the reaction kinetics may not favor this process in vivo. Nonetheless, there is not universal agreement regarding this proposed mechanism [46]. For example, an alternate proposal suggests that when ESH is oxidized by, for example, the hydroxyl radical, it can be “repaired” (i.e., regenerated) by interacting with vitamin C, which reduces oxidized ESH and simultaneously generates the ascorbyl radical [47]; of course, in cells, the ascorbyl radical would then need to be reduced [48]. In addition, thioredoxin reductase can directly reduce oxidized ESH by itself, without requiring GSH [49]. Finally, when human RBCs were subjected to oxidative stress using arsenicals, Reglinski et al. postulated a novel and unusual mechanism in which ESH may exert its protective effect by undergoing an “environmental”, but not a chemical, change, suggesting that it is not “consumed” when playing this role [50].

In contrast, Oumari et al. described an elegant mechanism, which does involve GSH, whereby ESH is regenerated after detoxifying singlet oxygen [35]. In addition, when ferryl (Fe^+4^) hemoglobin is produced by nitrite, it can be reduced by ESH, the thione of which is then “consumed” by forming the ESSE disulfide; ESH can then be regenerated (i.e., reduced) by interacting with thiol-containing cysteine and, perhaps, GSH [38]. Similarly, GSH may be involved in reducing oxidized ESH (i.e., the ESSE disulfide) after ESH participates in reducing ferryl (Fe^+4^) myoglobin [37].

Thus, there remains some debate whether, inside RBCs and other cells, oxidized ESH can be recycled without requiring reducing equivalents derived from, for example, the pentose phosphate pathway (i.e., NADPH). Therefore, it would be interesting to test this concept using RBCs from individuals with enzymatically diminished forms of glucose-6-phosphate dehydrogenase (i.e., G6PD), the rate-limiting step in the pentose phosphate pathway. Because G6PD-deficient RBCs cannot generate large amounts of NADPH, decreasing their ability to respond to oxidative stress because of defective regeneration of GSH, it would be interesting to determine whether or not oxidized ESH (i.e., ESSE disulfide) is efficiently regenerated in these RBCs.

### 3.3. How Does ESH Work in Cells and How Do Cells Modulate/Amplify Its Function(s)?

Using rats as a model organism, dietary supplementation with ESH protected kidney and liver tissues, and the polyunsaturated fatty acids (PUFAs) in their cellular membranes, from Fenton reaction-derived damage and lipid peroxidation [24]. In addition, ESH ameliorated iron-induced liver toxicity, perhaps by both scavenging ROS and binding iron [42]. Finally, in the setting of experimental diabetes, ESH protected rat endothelial cells against oxidative damage, thereby maintaining their normal function [52].

In the central nervous system, ESH improved antioxidant status (including the GSH/GSSG ratio) in the brains of mice [53]. In addition, ESH protected a human neuronal hybridoma cell line against hydrogen peroxide- and peroxynitrite-mediated toxicity, including preventing DNA damage [54]. Similarly, ESH inhibited hydrogen peroxide-mediated DNA damage and cell death in the PC12 rat neural cell line [55].

Interestingly, several studies suggest that one of the mechanisms, at least, by which ESH protects cells from oxidative damage is by upregulating signaling through the Nrf2 antioxidant pathway [56]. These studies examined oxidative damage induced by ultraviolet light in keratinocyte cell lines in vitro [57,58] and in a rat model of diabetes in vivo [59]. In addition, the latter provided evidence that ESH could also function by inhibiting inflammation mediated by the NFκB signaling pathway [59].

Given that a major effector of inflammation is the production of oxidant stress, it is perhaps not surprising that, in an effort to maintain homeostasis, inflammation could induce increases in intracellular ESH levels by enhancing its transport by SLC22A4, the highly specific ESH transporter (see just below). Indeed, *SLC22A4* mRNA levels in CD14+ cells were upregulated by proinflammatory cytokines, such as TNFα [60]. In addition, multiple pro-inflammatory signaling molecules, including, IL1β, TNFα, and NFκB, were each involved in upregulating SLC22A4 expression in human synoviocytes, which has potential relevance for the pathophysiology of rheumatoid arthritis [61].

## 4. How Does ESH Enter Organisms and Cells?

### 4.1. Identifying ESH Transporters and Their Cell/Tissue Locations

A major advance in the field of ESH biology was the discovery that the OCTN1 molecule (“Organic Cation Transporter-1”), which had previously been characterized as an “orphan“ transporter, putatively for small organic cations and zwitterions, was actually the highly specific cell membrane transporter of ESH [41]. Although this cell surface molecule had been given several names (e.g., OCTN1, ETT (“ergothioneine transporter”)), it is now generally agreed that its official name is SLC22A4 (i.e., “solute carrier family 22 member 4”); this is the descriptor we will use herein.

SLC22A4 protein and mRNA are highly expressed in various tissues, including the intestine [62], bone marrow [63], and fetal liver [63]. Nonetheless, there is some disagreement regarding whether or not it is highly expressed in adult liver [63,64]. Importantly, for the purposes of this review, in addition to its bone marrow expression, SLC22A4 is highly expressed by K562 cells [63], a human leukemic cell line that is often used to study the basic mechanisms involved in erythropoiesis [65].

More recent studies, which require further elaboration, suggest that a separate and distinct ESH transporter, SLC22A15, is required for ESH to enter the brain [66], even though relatively little ESH is found in mouse brain and SLC22A15 is less effective than SLC22A4 in transporting ESH [67]. In addition, the SLC22A15 transporter function is a bit more promiscuous, in that it can effectively transport carnitine and carnosine [66]. Although the tissue distribution of SLC22A15 overlaps with that of SLC22A4, it is much more prominent in the brain [66]. Nonetheless, studies suggest that no ESH is found in the brains of *SLC22A4* knockout mice (see below) [68,69]. Thus, more work remains regarding the biology of SLC22A15, and studies with single- and double-knockout (i.e., both *SLC22A4* and *SLC22A15*) mice could be very revealing.

### 4.2. Structure–Function Information about ESH Transporters

SLC22A4 is highly specific for transporting ESH and requires sodium ion to exert its effects [70]. Interestingly, analogous to ESH, carnitine, also a small zwitterion, is transported into cells by OCTN2, a highly homologous transporter, now officially designated as SLC22A5. Although SLC22A4 and SLC22A5 are highly homologous, they exhibit exquisite selectivity for ESH and carnitine, respectively, and recent, elegant molecular modeling studies helped explain these specificities [71]. In addition, site-directed mutagenesis studies of SLC22A4 and SLC22A5 were performed in an effort to help elucidate these specificities; however, none of the engineered SLC22A4 single point mutations allowed for carnitine transport; instead each maintained, or improved, ESH transport [72]. It is hoped that a complete elucidation of these specificities will become available in the future, which should also explain the apparent ability of SLC22A4 to transport metformin, among other small molecules [73]. Finally, at this time, less is known about the molecular mechanisms involved in ESH transport in the brain by SLC22A15 [66].

### 4.3. Elucidation of ESH Function Using Genetically Modified Organisms

The availability of *SLC22A4* knockouts in various model organisms has shed important light on ESH function and its biological importance. The first such model described *SLC22A4* knockout mice, in which ESH was not detected in any tissue examined [68]. In addition, although dietary ESH supplementation of these mice led to slight, transient increases in circulating ESH levels, no tissue incorporation was apparent. Given that few, if any, abnormalities were definitively detected in these mice at baseline, including no detectable hematological abnormalities, this suggests that an “ESH deficiency state” is not analogous to a vitamin deficiency. This concept is supported by an older study of rats fed an ESH-deficient diet, in that no abnormalities were seen in these rats or in their offspring, which were also maintained on an ESH-deficient diet [74]. Nonetheless, these knockout mice did exhibit greater intestinal susceptibility to oxidative stress. Indeed, a subsequent study of streptozotocin-induced diabetes in these mice identified increased oxidative stress and kidney fibrosis [75]. Thus, for the particular focus of this review, even though no hematological abnormalities were found in these mice at baseline, it is possible that they may have ineffective responses to disease-related stress. For example, crossing these mice with G6PD-deficient mice [76] might produce offspring exhibiting exacerbated oxidant-induced hemolysis (see below). Finally, although a SLC22A15 knockout mouse (i.e., Slc22a15^em1(IMPC)J^) is listed in the JAX catalog [77], to our knowledge, no papers have yet been published describing its biology. Nonetheless, studies with this mouse, along with those of a *SLC22A4/SLC22A15* double knockout, should be of interest.

Analogous ESH transporter constructs were also made in “simple” organisms, such as zebrafish [78] and roundworms [79]. Although these approaches will be useful for obtaining additional structure–function information and for elucidating the role(s) of ESH in the biology of organismal oxidative stress, there is currently no information regarding the effects of this “absolute” ESH deficiency on RBC biology in zebrafish, and *C. elegans* is not useful for this purpose.

### 4.4. Human ESH Transporter Variants

Multiple SLC22A4 polymorphisms were identified and characterized in human populations. Studying these naturally occurring variants is useful for elucidating transporter activity and substrate specificity [80,81], although their effects, if any, on RBC structure and function are not yet understood. In addition, there may be specific disease-relevant variants, which can influence intracellular ESH concentrations, such as in the context of Crohn’s disease [60]. Finally, although perhaps not relevant for RBC biology, SLC22A15 variants were also identified [66].

## 5. ESH and RBC Biology

### 5.1. Introduction

As described above, RBCs in vivo are continuously exposed to oxidant stress and, as such, use multiple mechanisms to resist these attacks and protect and/or repair oxidant damage to their cytoplasmic contents and cellular membrane [3,6]. From the perspective of transfusion medicine, refrigerated storage is necessary to maintain an appropriate, readily available inventory of donor RBC units so that they can be transfused into patients expeditiously, when required. During storage, these RBCs are exposed to additional oxidative stress, which, one can argue, is the “prime mover” in causing RBC “storage lesion” [82,83,84]. The latter encompasses a plethora of physiological and morphological effects [85,86], which ultimately lead to decreasing RBC storage quality and transfusion quality as the refrigerated storage interval increases; this results in individual patients receiving highly variable doses of therapeutically effective RBCs from individual donor units [87]. Interestingly, recent studies suggest that the “exposome”, as experienced by individual donors due to variations in diet, habits, pharmaceutical exposures, and the microbiome, may be quite relevant for the resistance or susceptibility of their donated units to the storage lesion [88]. Given that dietary supplementation with antioxidants can modulate the effects of the RBC storage lesion [84], and given that ESH is enriched in certain diets and was identified in the RBC exposome [88], it is not unreasonable to determine whether specific supplementation with ESH can affect RBC properties when circulating in vivo and during refrigerated storage in vitro, especially given ESH’s ability to interact directly with ROS and its possible interactions with free iron [9].

### 5.2. RBC ESH Levels

ESH, first identified more than a century ago, was also detected in the blood of humans and other animals not long thereafter [7]. Interestingly, the vast majority of “blood” ESH is found in RBCs (1285 ± 1363 ng/mL) [89], with only low levels in plasma (107.4 ± 20.5 ng/mL) [89], white blood cells, and platelets [7,68,90,91,92,93]. Indeed, *SLC22A4* knockout mice did not have any detectable ESH in their RBCs or plasma [68].

Multiple studies measured RBC ESH levels in various human populations and ethnicities, and in various disease settings [94,95]. Although RBC ESH levels vary significantly between individuals, there is little daily variation in serial measures in particular individuals [89,96]; nonetheless, it is possible to determine a reference range for humans [97]. Although RBC antioxidant potential appears to decrease with organismal aging [98], there is a lack of certainty whether this is also true for RBC ESH levels [10,96]; nonetheless, in rats, no change in RBC ESH levels was observed with aging [99]. Interestingly, ESH levels in RBCs decrease throughout their circulatory lifespan in vivo [100], perhaps contributing to their senescent phenotype by making them more sensitive to oxidative damage.

In an interesting study, when mice were given phenylhydrazine, a severe oxidant stressor that produces massive hemolysis, the RBCs remaining in the circulation had significantly higher ESH levels [76], perhaps suggesting that RBCs with low ESH levels were more susceptible to oxidant damage and were rapidly cleared. In addition, RBC ESH levels varied significantly when measured in RBCs obtained from genetically inbred strains of mice [101], suggesting that these levels may be genetically determined. Indeed, ESH levels were higher in RBCs from C57Bl/6 mice, as compared to FVB mice. This is particularly interesting given that, in the context of RBC storage and transfusion, C57Bl/6 mice are “good storers” and FVB mice are “poor storers” [101]; therefore, this difference in RBC ESH content may be physiologically relevant.

Multiple studies have evaluated the effects of dietary supplementation on ESH levels in “blood” in general, and in RBCs in particular. Given that the vast majority of ESH in the circulation is in RBCs, we assume that when “blood” levels were measured [22], these actually reflected ESH amounts in RBCs. For example, in a rat model, ESH accumulated slowly in circulating RBCs following dietary supplementation [102]. In addition, using radiolabeled ESH, the compound rapidly appeared in plasma and then disappeared rapidly, but then was stably present in circulating “corpuscles” with little or no decrease in cellular ESH levels after one week of fasting [23]. Analogous studies in rabbits showed that RBC levels decreased when they were fed an ESH-deficient diet, which then reversed with dietary supplementation [103]. Finally, in mice receiving one oral dose of radiolabeled ESH, RBC levels of this compound continued to increase slowly over the ensuing days [68]. Taken together, these results in animal models suggest that dietary ESH rapidly enters, and then rapidly disappears from, plasma; it also enters the RBC compartment slowly and then remains stable over time.

In humans, the failure to detect ESH in plasma from fasting subjects argues for its rapid clearance from this compartment, whereas the lack of an apparent effect of dietary ESH on steady-state RBC levels suggests that only persistent changes in dietary ESH uptake over long time periods would induce significant changes in RBC intracellular ESH concentrations [104]. Supporting this concept, a study of the effects of changes in altitude on human RBC metabolism demonstrated increasing 2,3-diphosphoglycerate levels with increasing altitude, presumably to enhance oxygen offloading during relative hypoxia; nonetheless, RBC ESH levels did not change, presumably resulting from a stable diet during the study period [105]. These data also suggest that RBC ESH was not “consumed” due to the altitude changes during this study.

Finally, compounds related to ESH, including trimethyl histidine (i.e., hercynine), methyl-ESH, and selenoneine are highly present in human RBCs [93]. Given the potential role of selenoneine as an antioxidant, and its abundance in certain foods (see above), it is surprising that mice fed a selenoneine-enriched diet for 32 days exhibited no detectable selenoneine in their RBCs [106]. Thus, more work on this interesting molecule is eagerly anticipated.

### 5.3. Does ESH Enter Mature RBCs or Only Erythropoietic Progenitors?

Some of the studies described above document that dietary ESH rapidly appears in plasma and is then rapidly cleared, over minutes to hours; it then appears slowly in circulating RBCs, over days to weeks, and remains relatively stable in this compartment. These results are consistent with the concept that ESH enters the RBC compartment during erythropoiesis, but does not enter into, or efflux from, mature circulating RBCs. These results would also be consistent with the absence of functional SLC22A4 transporters, or the ability of these molecules to function effectively, on the surface of mature RBCs. They also suggest that there is little or no efflux out of mature RBCs, and, therefore, any changes in RBC levels would be due to intracellular “consumption” in response to oxidative stress. Data directly supporting, or refuting, this hypothesis are not entirely consistent and are described just below.

Although several studies support the concept that ESH can be transported into mature RBCs [92,107], other, possibly more convincing, studies demonstrate that ESH does not directly enter into, or efflux out of, mature RBCs [41,67,91]. Indeed, the functional expression of SLC22A4 was not detected on mature RBCs and reticulocytes, suggesting that ESH found in RBCs derives from its transport into erythropoietic progenitors, remaining there throughout their differentiation into mature RBCs [104]. To this end, erythropoietic progenitors express high levels of *SLC22A4* mRNA [41,107]. In addition, *SLC22A4* mRNA is detectable in human hematopoietic stem cells [41] and may be relevant in the context of polycythemia vera [108]. In addition, given that different types of reticulocytes are identifiable, and that they may vary in their antioxidant “machinery” [5], it would be interesting to evaluate *SLC22A4* mRNA and ESH levels in these cell types, particularly in disease settings that induce stress erythropoiesis. Finally, given the increasing sensitivity in identifying additional components of the human and murine RBC proteome [109], it would be important to determine definitively, using highly purified samples of mature RBCs, lacking leukocytes and platelets, whether or not the SLC22A4 transporter protein is present and functional on these cells.

An alternative approach to address this issue could use tissue culture in vitro. For example, the immortalized K562 human cell line expresses high levels of *SLC22A4* mRNA [63] and can be induced to differentiate into RBC-like, hemoglobin-containing cells [65]. In addition, silencing *SLC22A4* decreases both transporter expression and ESH uptake [110]. Thus, it would be interesting to measure SLC22A4 protein levels and transporter function in terminally differentiated K562 RBC-like cells. Analogous approaches could also be employed using mouse or human CD34+ hematopoietic stem cells, derived from various sources, by culturing them in vitro and inducing them to differentiate into, at least, enucleated reticulocytes [111].

### 5.4. What Are the Functions of ESH in RBCs?

Given the high amounts of ESH in circulating mature RBCs, and the expression of its transporter in the bone marrow and during erythropoiesis [63], it is reasonable to suggest that the major function of ESH is to protect erythropoietic progenitors and mature RBCs from oxidative stress induced by hemoglobin auto-oxidation. Indeed, silencing *SLC22A4* mRNA expression in K562 cells induces decreased expression of the transporter protein, decreased cellular uptake of ESH, and decreased proliferation and differentiation of these cells, potentially due to increased apoptosis [110]; these results suggest a role in protecting erythropoiesis from damaging oxidative stress. In addition, in rabbits fed an ESH-deficient diet, RBC ESH levels decreased, and these RBCs were more sensitive to sodium nitrite-induced methemoglobin formation; importantly, this defect was reversed by dietary supplementation with ESH [103]. Similar results for met-hemoglobin production were seen in rats fed a methionine-deficient diet; in addition to observing lower ESH levels, ESH was found to be more important than reduced glutathione for providing this protection [112]. Although ESH was also reported to protect human RBCs from oxidative damage due to arsenicals [50], some questions were raised about these results [113].

If, indeed, RBC ESH levels decrease with human aging, this may enhance their “senescent phenotype”, which could potentially be ameliorated by dietary ESH supplementation [10]. Nonetheless, despite *SLC22A4* knockout mice having no detectable ESH in their circulating RBCs, they were not anemic, nor did they display hematological abnormalities suggestive of dysfunctional hematopoiesis [68]. However, these results did not derive from intensive studies of murine erythropoiesis in vivo or in vitro, nor were these animals subjected to a disease model (e.g., sepsis) that would induce stress erythropoiesis. As such, it would be interesting to breed these mice with those exhibiting an RBC disease phenotype of increased oxidative stress (e.g., G6PD deficiency, sickle cell disease), to study the relevance, or lack thereof, of ESH function in these disease contexts.

### 5.5. Clinical Implications of ESH in RBC Biology

Due to its antioxidant properties and apparent safety as a dietary supplement, increasing numbers of publications have suggested the potential benefits of ESH supplementation in various non-hematological disease settings, and as an anti-aging nutraceutical (for a general review, see [8]). In addition, in the context of non-hematological disorders, circulating RBCs can function as sensors, measuring and integrating oxidative stress throughout a given organism [3]. As such, measuring RBC ESH levels could identify a potential organismal deficit (or abundance) of antioxidant potential. As one example, RBC ESH levels were significantly elevated in patients with rheumatoid arthritis [60]. Because pro-inflammatory cytokines and cytokine signaling are prominent in this disease (e.g., IL1β, TNFα, NFκB) [114], and because these increase SLC22A4 expression in synoviocytes [61], this may provide a compensatory mechanism to provide protection by increasing ESH levels in these cells. If these cytokines similarly affected bone marrow erythropoiesis by increasing SLC22A4 expression and function, then elevated circulating RBC ESH levels could provide a marker of disease activity. Similarly, in pre-eclampsia [115], the pathogenesis of which may also involve oxidative stress [116], this proposed mechanism could also explain the presence of elevated RBC ESH levels, perhaps as part of a compensatory process [117]. Finally, it is important to consider the possibility that elevated ESH levels in various tissues, including RBCs, may exacerbate cancer and certain infections by ameliorating the oxidative stress with which the host could target the pathogenic process [8]. Thus, it is important to consider this potential adverse effect if a therapeutic approach aims to use dietary supplementation to raise RBC ESH levels.

In contrast, in the context of hematological disorders, ESH levels may modify disease severity in settings where RBCs are particularly susceptible to oxidative damage, thereby leading to intravascular and/or extravascular hemolysis. For example, low RBC ESH levels may exacerbate hemolytic crises in patients with G6PD deficiency or shorten RBC circulatory lifespan in patients with sickle cell disease. Nonetheless, it is important to note that, following a hemolytic crisis in G6PD deficiency, the remaining RBCs are typically younger, with elevated reticulocyte counts; both of these cell types have higher than average G6PD enzymatic activity (in the context of the average activity of G6PD-deficient RBCs) because it typically decreases during circulatory aging in vivo [118]. Similarly, RBC ESH levels presumably decrease throughout their circulatory lifespan [100]. Interestingly, although ESH was not identified in RBCs in a metabolomics investigation of sickle cell disease [119], it was found to be significantly decreased in an analogous study [120]; the authors of the latter suggested that dietary ESH supplementation could be therapeutically beneficial for these patients [120].

Although not a hematological disorder per se, experimental hemorrhage in a rat model led to increased RBC ESH levels following recovery [99]. Similar results were seen in humans in the more controlled and limited context of blood donation (see below) [94]. A potential explanation for this finding could be that erythropoietic recovery yields increased numbers of circulating (stress) reticulocytes and younger RBCs, which can have higher ESH levels in their cytoplasm.

Finally, the RBCs of patients with hereditary overhydrated stomatocytosis, due to variants in Rh-associated glycoprotein, have low ESH levels [121]; similar results were found in patients with chronic granulocytic leukemia [122]. In addition, erythropoietic progenitors in patients with polycythemia had an increased expression of *SLC22A4* mRNA [108]. Nonetheless, the mechanistic explanations for these results, and their pathologic implications, are not yet known.

### 5.6. Clinical Applications of ESH for RBC Transfusion and the RBC Storage Lesion

From the perspective of volunteer blood donation, acute phlebotomy of ~10% of a donor’s circulating blood volume subsequently induces enhanced erythropoiesis in an effort to restore RBC mass to baseline levels. Assuming that there is sufficient ESH in the diet, one might predict that RBC ESH levels, on average, might increase, as a result of this burst of newly formed RBCs. Indeed, in an early study of healthy human volunteers, RBC ESH levels did increase at two weeks post-donation [94].

Following donation, human RBC concentrates, which can be refrigerator-stored for up to 42 days before transfusion, experience the “storage lesion”, which is primarily due to oxidative stress (see above). Interestingly, in an early study using a rabbit model, RBC ESH levels decreased during storage [103]. In contrast, in a mouse model, RBC levels remained stable, or even increased, during storage [101]. However, whether or not ESH levels change during the storage of human RBCs is not yet known. Nonetheless, given the potential role of ESH in ameliorating oxidative stress during storage, thereby decreasing the severity of the storage lesion, one might expect that RBC ESH levels, and/or *SLC22A4* variants, would correlate with RBC transfusion quality (e.g., post-transfusion hemoglobin increments); however, no evidence for this effect has been described to date [87,123]. Nonetheless, results from mouse studies (see above) demonstrate differences in RBC levels when comparing inbred strains; in particular, levels in C57Bl/6 RBCs were significantly higher than those in FVB RBCs [101]. Given that FVB and C57Bl/6 mice are “poor storers” and “good storers”, respectively [101], and that their respective *SLC22A4* sequences differ somewhat, this is an intriguing hypothesis to pursue.

## 6. Conclusions

In summary, ESH, an unusual and interesting antioxidant, is obtained from the diet, through the mediation of a highly specific and evolutionarily conserved transporter, and is present in high amounts in circulating mature RBCs. Although there are no known deficiency states requiring its definition as a “vitamin”, there is increasing evidence that ESH could be therapeutically beneficial as a “nutraceutical”. Given that oxidant stress is important in the pathophysiology of multiple hematological disorders, and is a major determinant of RBC lifespan in vivo and in vitro, there not only remains much to be learned regarding ESH function during erythropoiesis and in RBC biology, but there also is the potential that it could enhance the function, health, and longevity of human RBCs (Table 1).

## Figures and Tables

**Table 1:** Unanswered questions.

**At which developmental stages during erythropoiesis can ESH be imported into cells?**
Can use ESH supplementation at different developmental stages during differentiation of K562 cells or CD34+ cells in vitro.
2. **How do *SLC22A4* gene transcription, SLC22A4 protein expression, and SLC22A4 transport function change during erythropoiesis?**
Can use highly sensitive proteomics methods and differentiation of CD34+ cells in vitro, to determine whether the transporter, or its fragments, can be identified at different cell stages.
3. **How do RBCs “normally” accumulate ESH? Can ESH enter mature RBCs circulating in vivo and/or “in the bag” in vitro?**
Can incubate isotopically labeled ESH in vitro (using radioactive or stable isotopes) with highly purified populations of reticulocyte-poor, mature human and mouse RBCs. Can use ESH dietary manipulation of mice, along with RBC biotinylation in vivo, to label and then isolate RBC populations of defined circulatory age.
4. **Do *SLC22A4* sequence variants affect the “efficiency” of ESH uptake during erythropoiesis (and/or in mature RBCs), thereby affecting RBC ESH levels?**
Can study RBCs obtained from human volunteers with *SLC22A4* sequence variants, inbred mouse stains with known SLC22A4 sequence variants, or genetically modified mice constructed to express specific *SLC22A4* sequence variants of interest. Can study differentiation of CD34+ cells obtained from human volunteers with *SLC22A4* sequence variants.
5. **Does “stress” and/or disease modulate RBC ESH levels?**
Does organismal “stress” in vivo (e.g., sepsis) or cellular stress in vitro (e.g., endotoxin exposure) affect SLC22A4 transporter levels and/or function and RBC ESH levels? Are there differential effects on reticulocytes, stress reticulocytes, and/or mature RBCs in hematological disorders, such as sickle cell disease? Do responses to anemia per se (e.g., hemorrhage resuscitation, iron therapy to treat iron-deficiency anemia, repetitive phlebotomy, and blood donation in healthy volunteers) affect SLC22A4 expression and function during erythropoiesis and ESH levels in mature RBCs? Does inflammation in previously healthy individuals, or in patients with inflammatory disorders, affect SLC22A4 expression and function during erythropoiesis and ESH levels in mature RBCs?
6. **Is decreased SLC22A4 function, resulting in decreased RBC ESH levels, relevant in hematological settings?**
Do *SLC22A4* knockout mice display any hematological pathology and/or defective erythropoiesis when placed under stress (e.g., inflammation, sepsis, hemorrhage resuscitation, iron deficiency, or immune-mediated hemolysis)? Does a defective SLC22A4 function (e.g., by knocking out *SLC22A4* expression) exacerbate hematological disorders, such as G6PD deficiency and sickle cell disease? Does knocking out both ESH transporters (i.e., SLC22A4 and SLC22A15) produce any additive effects in the settings describe above?
7. **Do increased RBC ESH levels provide enhanced protection against oxidative stress in settings of NADPH “restriction” (e.g., G6PD deficiency)?**
8. **Are RBC ESH levels independently relevant for modulating RBC “circulatory quality” (e.g., RBC lifespan, deformability, endothelial adherence, perfusion, oxygenation, and erythrophagocytosis)?**
9. **What roles, if any, do RBC ESH levels play in refrigerated storage biology?**
Do RBC ESH levels modulate the RBC storage lesion, thereby affecting RBC “storage quality”? Do *SLC22A4* variants in RBC donors affect RBC ESH levels, RBC “storage quality”, and post-transfusion hemoglobin increments? If healthy blood donors are supplemented with dietary ESH, do their RBCs exhibit enhanced “circulatory quality” post-transfusion?
